# Polymorphisms in *MC1R* and *ASIP* Genes are Associated with Coat Color Variation in the Arabian Camel

**DOI:** 10.1093/jhered/esy024

**Published:** 2018-06-09

**Authors:** Faisal Almathen, Haitham Elbir, Hussain Bahbahani, Joram Mwacharo, Olivier Hanotte

**Affiliations:** 1 Department of Veterinary Public Health and Animal Husbandry, College of Veterinary Medicine, King Faisal University, Saudi Arabia; 2 The Camel Research Center, King Faisal University, Saudi Arabia; 3 The Department of Biological Sciences, Faculty of Science, Kuwait University, Safat, Kuwait; 4 The International Centre for Agricultural Research in the Dry Areas (ICARDA) c/o ILRI-Ethiopia Campus, Addis Ababa, Ethiopia; 5 The School of Life Sciences, University of Nottingham, University Park, Nottingham, UK; 6 LiveGene, International Livestock Research Institute, Addis Ababa, Ethiopia

**Keywords:** Arabian camel, dromedary, Camelidae, pigmentation, SNPs

## Abstract

Pigmentation in mammals is primarily determined by the distribution of eumelanin and pheomelanin, the ratio of which is mostly controlled by the activity of melanocortin 1 receptor (MC1R) and agouti signaling protein (ASIP) genes. Using 91 animals from 10 Arabian camel populations, that included the 4 predominant coat color phenotypes observed in the dromedary (light brown, dark brown, black, and white), we investigated the effects of the *MC1R* and *ASIP* sequence variants and identified candidate polymorphisms associated with coat color variation. In particular, we identified a single nucleotide polymorphism (SNP), found in the coding region of *MC1R* (901C/T), linked to the white coat color, whereas a 1-bp deletion (23delT/T) and a SNP (25G/A) in exon 2 of *ASIP* are associated with both black and dark-brown coat colors. Our results also indicate support that the light-brown coat color is likely the ancestral coat color for the dromedary. These sequence variations at the *MC1R* and *ASIP* genes represent the first documented evidence of candidate polymorphisms associated with Mendelian traits in the dromedary.

## Introduction

Livestock populations and breeds display large diversity in coat colors and patterns, with coat color being most often a breed defining trait. Coat color variation has been associated with adaptation to environmental pressures (e.g. natural selection following predation and/or thermoregulation (Finch et al. 1984)). In particular, it is recognized that dark-coated animals absorb more heat from solar radiation than light-coated ones (Finch et al. 1984). Accordingly, during daytime it may be advantageous to be dark-coated in cold environments but light-coated in hot areas (Finch 1981). In this respect, dark-coated animals living in desert environments, which are characterized by large diurnal temperature differences, may be advantageous (Ward et al. 2002). Connection between coat color and productivity has also been suggested (e.g. black-coated goats gaining weights much faster, due to reduced metabolic rates, compared with white coated ones (Singh et al. 1997)).

The discovery and analysis of candidate coat-color–associated genes have led to a better understanding of the mechanism of pigmentation in mammalians. More than 150 coat-color–associated genes have been identified in mammals with or without epistatic interactions (Cieslak et al. 2011). Amongst these, melanocortin 1 receptor (*MC1R*) and agouti signaling protein (*ASIP*) genes are commonly associated with coat color variation. Indeed, pigmentation in mammals is primarily determined by the distribution of eumelanin (dark brown to black) and pheomelanin (yellowish to reddish), the ratio of which is mostly controlled by the activities of *MC1R* and *ASIP* proteins (Jackson 1994). Both eumelanin and pheomelanin are synthesized in the melanocytes via melanogenesis. Melanocyte stimulating hormone (*MSH*) activates *MC1R*’s melanogenic activity. This leads to an increase in cyclic adenosine monophosphate (cAMP) protein, resulting in enhanced tyrosinase production and the switching of the signaling pathway from pheomelanin to eumelanin (Garcia-Borron et al. 2005). In contrast, *ASIP* plays a primary role in increasing the production of pheomelanin rather than eumelanin by inhibiting the *MC1R* signaling pathway (Ha et al. 2003). Other proteins, involved in the same pathways, include tyrosinase-related protein 1 (*TYRP1*) and membrane-associated transporter proteins (*MATPs*) with mutations affecting coat color (Wang and Hebert 2006).

Loss-of-function mutations in the *MC1R* gene lead to a switch to the pheomelanin synthetic pathway. Such dysfunctional mutations have been associated with light-coated animals, such as the white Kermode bear (Ritland et al. 2001), the yellow labrador, the golden retriever (Everts et al. 2000), and the chestnut-colored horses (Marklund et al. 1996). Contrastingly, loss-of-function mutations in the *ASIP* gene give rise to dark-colored animals in the presence of functional alleles of the *MC1R* gene, as the coloration is primarily determined by eumelanin. This is exemplified in the black Xalda sheep (Royo et al. 2008), the recessive black plumage in Japanese quail (Hiragaki et al. 2008) and the dark pigmentation of the standard silver fox (Vage et al. 1997).

The genetic control of coat color in the dromedary *Camelus dromedarius* remains unknown. A molecular genetic study in Pakistani dromedary populations identified a SNP at position 901 (C/T) in the coding region of the *MC1R* gene and 2 SNPs at positions 113 (C/T) and 200 (C/T) in exon 1 of the *TYRP1* gene (Qureshi et al. 2008). None of these mutations was, however, linked to any particular coat color. In alpaca *Vicugna pacos*, molecular studies have shown that *MC1R* and *ASIP* genes are the main candidates determining coat color (Cransberg and Munyard 2011; Chandramohan et al. 2013). The studies identified 2 linked missense mutations (positions 82 A/G and 901 C/T) in the coding region of the *MC1R* gene in animals lacking black pigmentation, while 3 mutations (2 missense mutations and a deletion) in exon 4 of the *ASIP* gene were associated with black coat color (Feeley and Munyard 2009; Feeley et al. 2011). No candidate mutation has so far been detected in *TYRP1* and *MATP* genes in alpaca (Cransberg and Munyard 2011).

**Figure 1.**
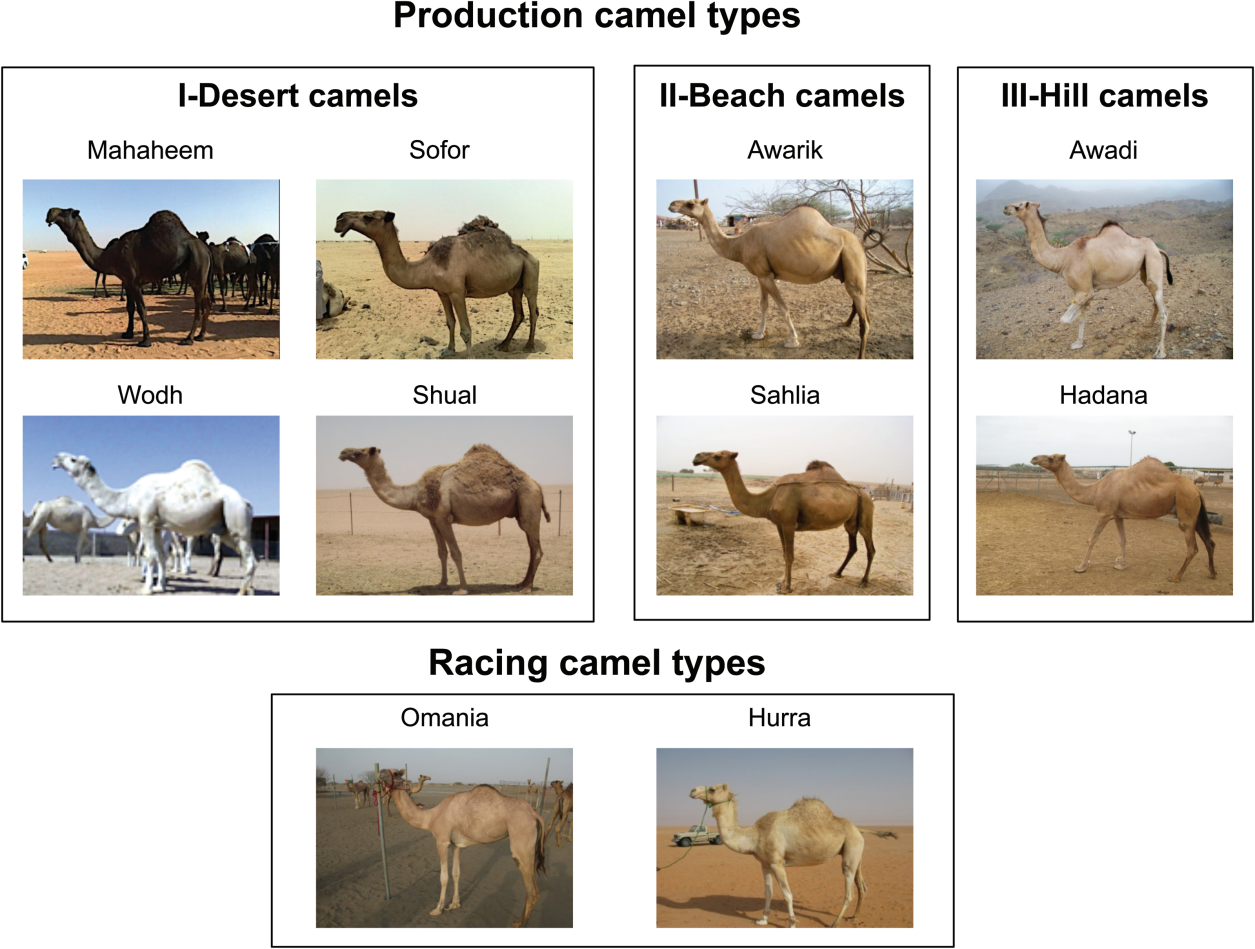
The different types of Arabian Peninsula dromedary; following Abdallah and Faye (2012) and Al-Eknah (personal communication).

In contrast to other livestock species, coat color in dromedary is typically uniform, with the exception of spotted color patterns observed in some West African dromedary (Traore et al. 2014). It varies from a completely black (e.g. Magaheem dromedary) to a completely white (e.g. Wodh dromedary), with the intermediate brown-colored phenotypes being the commonest (Figure 1). The classification of Saudi Arabian camel is unclear. It has been suggested to separate the Saudi Arabian camels into 3 ecotypes based on their distribution (desert, hill, and beach), with the racing camels representing a separate group (Abdallah and Faye 2012; Al-Eknah, personal communication; Figure 1). These ecotypes are characterized to some extent by the differences in morphology (body conformation), hair structure, and coat color (Abdallah and Faye 2012; Figure 1). In this study, using prior knowledge from alpaca indicating the importance of *MC1R* and *ASIP* genes in coat color variation, we characterized the variability in the coding region(s) of *MC1R* and *ASIP* genes to ascertain the potential effects of the 2 genes on coat color variation in the dromedary.

## Materials and Methods

### Sample Collection

Ninety-one unrelated animals from ten populations of Arabian Peninsula dromedaries (Table 1; Figure 1; Almathen 2014) were studied. The Magaheem dromedaries are predominantly black, although combinations of black and dark-brown coats can be observed. They originate from the central southern part of Saudi Arabia, within and surrounding the ar Rub’ al Khali desert (the Empty Quarter Desert). They are known as the camels of the Al Dawasir and Al Murrah tribes. The Wodh dromedaries are white or light cream in color. Together with Sofor, Hurra, and Shual dromedary populations, they are largely found in the northern part of the Arabian Peninsula. The Sofor dromedaries are dark brown on the shoulder, hump, and tail. These distinguishes them from the Hurra and Shual dromedaries, which are characterized by a light-brown coat. The other Arabian Peninsula dromedary populations (Sahlia, Hadana, Awadi, Awarik, and Omani) are typically light brown (Figure 1). The Omani dromedary population originated from the southwest of the Arabian Peninsula, whereas the Sahalia, Hadana, Awadi, and Awarik dromedaries are from the southeast of the Arabian Peninsula. During sampling, digital images were taken and coat colors were recorded for the 91 animals. For the purpose of this study, we classified the coat colors into 4 groups based on the presence or absence of black color and the intensity of brown color *viz*: 1) light brown (*n* = 30), 2) dark brown (*n* = 18), 3) black (*n* = 22), and 4) white (*n* = 21) (Table 1; Figure 1). Whole blood was collected from the 91 animals and spotted on FTA® cards (Whatman Inc., NJ).

**Table 1:** Sample size and coat color phenotypes of the 10 Arabian Peninsula dromedary populations

Coat color group	(Number)	Population	Sample size
Black	(*n* = 22)	Magaheem	22
Dark brown	(*n* = 18)	Sofor	15
		Magaheem	3
Light brown	(*n* = 30)	Omania	5
		Awarik	8
		Awadi	3
		Shual	3
		Sahlia	3
		Hadana	3
		Hurra	5
White	(*n* = 21)	Wodh	21
**Total**	**91**		**91**

### DNA Extraction, Amplification, and Sequencing

Genomic DNA was extracted from blood spotted on FTA® cards using a modified protocol (available from the corresponding author on request) following Smith and Burgoyne (2004). The coding regions of *MC1R* and *ASIP* genes were amplified using published primers designed for alpaca (Feeley and Munyard 2009; Feeley et al. 2011; see Supplementary Table S1), whilst for exon 4 the forward primer sequence was modified according to the Arabian *Camelus dromedarius* reference genome assembly (GenBank accession number GCA_000767585.1) (Wu et al. 2014; see Supplementary Table S1). All PCR reactions were carried out in a total volume of 25 µL containing 5–20 ng genomic DNA, 0.5 µM of each primer, 1 unit of GoTaq® polymerase (Promega, UK), 0.2 mM of each dNTP (Qiagen), 10X GoTaq Flexi Buffer, and 1 mM MgCl_2_ (Promega). PCR cycles included an initial denaturation step at 95 °C for 2 min, followed by 35 cycles, each consisting of denaturation at 95 °C for 40 s, annealing at various temperatures depending on the primers (see Supplementary Table S1) for 45 s and extension at 72 °C for 35 s, and a final extension step of 5 min at 72 °C. PCR products were purified using the QIAquick^®^ PCR Purification Kit (Qiagen) following the manufacturer’s instructions; they were sequenced at Macrogen (http://www.macrogen.com/) using the original PCR forward and reverse primers. Sequences were manually inspected and aligned using BioEdit v. 5.0.6. The position and number of SNPs were assigned with Geneious v. 6.1.7 (Kearse et al. 2012) using the alpaca *MC1R* (GenBank accession number: EU135880) and *ASIP* (GenBank accession number: HQ645015 and HQ645017) sequences as references. We also used the Bactrian camel (*Camelus bactrianus*) *MC1R* (GenBank accession number: AB495001) and the wild Bactrian camel (*Camelus ferus*) *ASIP* (GenBank accession number: XM_010960842.1) sequences for comparison purposes. The *MC1R* sequences generated in this study have been deposited with the GenBank under accession numbers: KU179867 and KU179868.

### Statistics Analysis

Differences in allele frequencies between coat color groups (white versus nonwhite) were tested using a chi-square test in XLSTAT (www.xlstat.com/en/).

## Results

**Figure 2.**
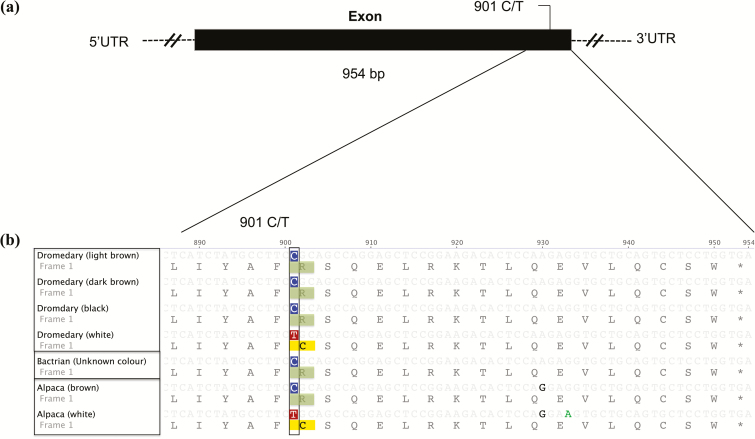
The dromedary melanocortin 1 receptor *(MC1R*) gene. (a) The exon region is shown as a solid black box; un-translated regions are shown as broken lines (not drawn to scale). Single nucleotide polymorphisms are shown in the region where they occurred. (b) Nucleotide and amino acid sequence comparisons for the last segment of the coding region for the 4 defined dromedary coat color groups and other camelids. Polymorphisms are shown inside the nonsolid box.

A sequence of about 1100 bp including the coding region and parts of the 5′- and 3′-un-translated regions (UTR) of the *MC1R* gene was successfully amplified in the 91 dromedaries. The complete coding region of the dromedary *MC1R* gene is 954 bp long and it encodes a protein of 317 amino acids. Alignment with the alpaca and Bactrian camel reference sequences showed 98% and 99% sequence homology, respectively (see Supplementary Figure S1). A single SNP (exon position 901, C/T; Figure 2a) encoding a missense mutation (nonsynonymous) was found in the dromedary. This polymorphism results in an amino acid change from arginine (R) to cysteine (C) at protein position 301 (Figure 2b). All nonwhite individuals (black, dark, and light brown, *n* = 70) were homozygous for the 901C allele, whereas all white dromedaries (Wodh population, *n* = 21) were either homozygous for the 901T (*n* = 8) or heterozygous (*n* = 13) for the 901C/T alleles (Tables 1 and 2) (*P* < 0.0001, df = 1).

**Table 2:** Association between the 4 defined dromedary coat color groups and polymorphism at the coding region of melanocortin 1 receptor (MC1R) and exon 2 of agouti signaling protein (ASIP) genes

Coat color group	MC1R	ASIP (Exon 2)	
	901 bp position	23 bp position	25 bp position
Black and Dark brown	C/C	^Del^T/^Del^T	A/A
Light brown	C/C	T/T	G/G
White	C/T or T/T	T/T	G/G

**Figure 3.**
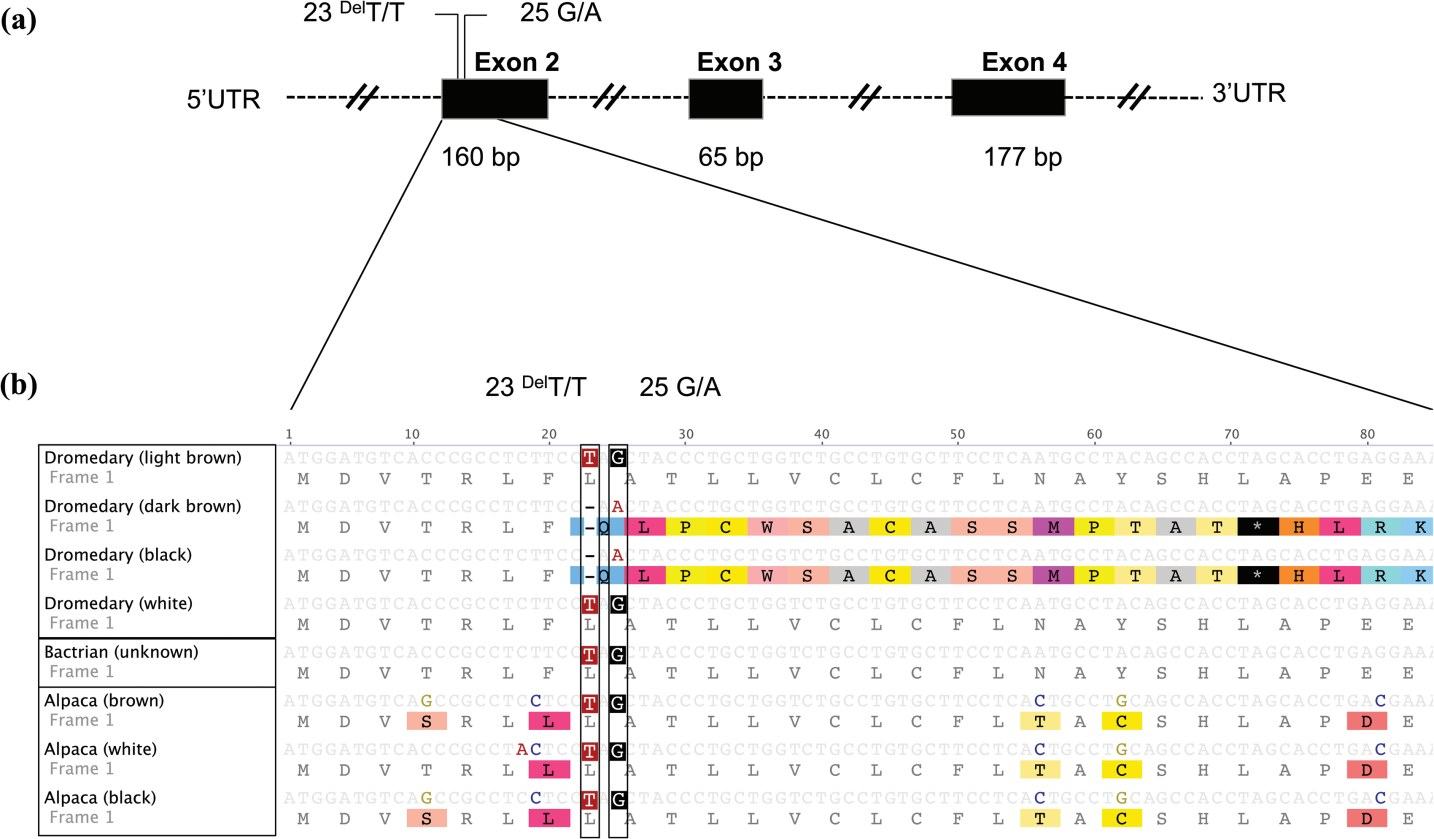
Agouti signaling protein *(ASIP*) gene sequences according to the alpaca (Feeley et al. 2011). (a) The exons are shown as solid black boxes; un-translated regions are shown as broken lines (not drawn to scale). Single nucleotide polymorphisms (SNPs) are shown in the regions where they occurred. (b) Nucleotide and amino acid sequence comparisons for exon 2 for the 4 defined dromedary coat color phenotypes and other camelids. Polymorphisms are shown inside the nonsolid box.

We successfully amplified and sequenced exons 2, 3, and 4 of the *ASIP* gene in the 91 animals (Table 2). The dromedary exon 2 sequence was found to be 160 bp long and it shows a 98% and 100% homology with the alpaca and the wild Bactrian camel reference sequences, respectively (see Supplementary Figure S2). Two polymorphisms were identified in exon 2 of the dromedary, a 1-bp “T” deletion (23^del^T) and a SNP (G/A) at position 25 (Figure 3a). These 2 mutations define 2 haplotypes and 2 homozygous genotypes, 23^del^T-25A and 23T-25G. The 23^del^T-25A haplotype results in a frame shift mutation, which changes the amino acid sequence and introduces a premature stop codon at position 24 (Figure 3b). Animals with black and dark-brown coat colors (*n* = 40, Magaheem and Sofor) were homozygous for haplotype 23^del^T-25A, whereas all light-brown and white individuals (*n* = 51) were homozygous for the 23T-25G haplotype (Tables 1 and 2). No polymorphisms were detected in exons 3 and 4.

To infer the possible ancestral wild-type alleles of these polymorphisms, we compared our sequence variants with those of the domestic Bactrian camel and alpaca. For *MC1R*, we found that the 901C allele is present in the brown coat colored alpaca (GenBank accession number: FJ847233) and in the Bactrian camel (GenBank accession number: AB495001), whereas the 901T allele is associated with the white coat alpaca sequence (GenBank accession number: FJ847232; Figure 2a). Comparing the sequences from exon 2 of *ASIP*, we found that the 23T-25G haplotype is present in the reference alpaca and the wild Bactrian camel sequences (GenBank accession numbers: HQ008273 and XM_006190779, respectively); no deletion was identified in both species (Figure 3b).

## Discussion

Candidate polymorphisms at *MC1R* and *ASIP* genes associated with coat color variation have been reported in several species including alpaca (Feeley and Munyard 2009; Feeley and Munyard 2011; Cransberg et al. 2013). Here, we assessed the presence of polymorphisms at these 2 genes and their putative association to coat color variation in the Arabian camel. Analysis of the coding region sequences of *MC1R* and exon 2 of *ASIP* supports key roles of these 2 genes in the genetic control of coat color in the dromedary. Our data indicate that the homozygous 901C allele at the exon of the *MC1R* gene and haplotype 23T-25G in the exon 2 of the *ASIP* gene probably represent the ancestral wild types in the dromedary. They are found in all light-brown dromedaries and other camelids (Figures 1 and 2), with also the majority of dromedaries today being light brown (Abdallah and Faye 2012). The homozygous 901C allele was found in all the Arabian dromedary populations, except in the Wodh population (white dromedaries), whereas the 23T-25G haplotype was found in all populations, except the dark-brown or black-coated Magaheem and Sofor dromedaries.

The protein *MC1R* acts as a genetic switch that determines whether dark eumelanin or light pheomelanin is produced in regulating coat color. α-MSH, a melanocyte stimulating hormone, binds to the *MC1R* receptor triggering eumelanin production. It competes with *ASIP*, which also binds to the *MC1R* receptor to switch over the production of pheomelanin (Klungland et al. 1995). In the dromedary, it seems that the functional *ASIP* ancestral wild-type alleles (23T-25G) regulate the expression of the *MC1R* gene in such a way that it reduces the deposition of eumelanin resulting in brown coat color. Although most dromedary populations have brown coats, its intensity varies within and between populations. It is possible that such variation is the consequence of epistatic interactions with other gene products besides those from *MC1R* and *ASIP* genes. Also, the structure analysis of the coding region of *MC1R* indicates that the change in amino acid from arginine to cysteine occurs in the C-terminal end of the *MC1R* protein, which comprise the last 17–19 amino acids in most animals (Garcia-Borron et al. 2005). The *MC1R* C-terminal peptide region is essential for proper gene function, particularly in protein structure functional coupling efficacy (Sanchez-Mas et al. 2005).

Mutations at the *MC1R* gene have been associated with the white coat phenotype in several other mammalian species. The same 901C/T polymorphism was described in alpaca where the 901T allele and a linked polymorphism (82G) were associated with the absence or reduction in eumelanin pigmentation (Feeley and Munyard 2009), further supporting a key role for this gene in determining white coat color in Camelidae. Interestingly, the level of variation at the *MC1R* coding region is very low in dromedary with only 1 polymorphism (901C/T) compared with 21 reported in alpaca (Feeley and Munyard 2009). However, in this later species, not all these polymorphisms are likely associated with coat color phenotypes. Such level of polymorphisms may translate to a larger founding ancestral population for today’s alpaca compared with the dromedary. This remains to be further investigated. Polymorphisms at nucleotide position 901 of the *MC1R* gene have also been associated with coat color variation in noncamelids. In horses, the *MC1R* 901C/T polymorphism has been linked to the chestnut coat color (Marklund et al. 1996; Rieder et al. 2001). Analysis of this mutation in alpacas and horses show the 901T allele to be recessive (Rieder et al. 2001; Feeley and Munyard 2009). Although the pedigree information is lacking here, our findings seem to suggest that this allelic state is dominant in the dromedary over the 901C one as heterozygous animals (901C/T) display a white coat color. A dominant allele at the *MC1R* gene that is associated with the white coat color phenotype has been reported in White leghorn chickens (Kerje et al. 2003). The complete absence of black and brown coat colors in animals with the 901T genotype suggests that this mutation leads to a complete loss-of-function of the *MC1R* gene. It is possible that the 901T allele results in the failure of the α-MSH to properly bind to the *MC1R* receptor, resulting in a loss of activation of the receptor preventing the initiation of the eumelanin synthesis. Nevertheless, further investigations on the functional significance of the 901T allele and its possible interactions with other genes would be necessary to evaluate the role of the *MC1R* gene in white pigmentation in the dromedary.

We found that the black and dark-brown coat colors in the dromedary are probably caused by the 23^del^T-25A haplotype in exon 2 of the *ASIP* gene, resulting in a complete loss-of-function, as it results in a premature stop codon (Figure 3b). These mutations have not been identified, so far, in other mammalian species, including other camelids. However, a deletion in exon 2 causing loss-of-function in the *ASIP* gene appears to be common in mammalian species (e.g. sheep (Fontanesi et al. 2011), horses (Rieder et al. 2001), and cats (Eizirik et al. 2003)). These mutations have been shown to be associated with a recessive black coat color in these 3 species. It remains unclear why we did not observe any heterozygote individuals with the combinations (23T-25G/23^del^T-25A); pedigree analysis is therefore required to assess the Mendelian inheritance and segregation pattern of this phenotype in the dromedary.

Regarding the possible environmental adaptation and/or human selection of the different dromedary coat color phenotypes, it is worth noting that the dark-brown, black, and white coats are not found in hill and beach dromedary ecotypes, whereas all the 4 coat colors occur in populations of dromedary living in the desert (Almathen 2014). Such diversity of coat colors in the latter may be an adaptive trait, considering the diversity of the desert environments. The Magaheem population originates from the Empty Quarter desert, the largest continuous expanse of sand desert in the world (Vincent 2008). The brown and white coat color dromedaries are found in the northern part of the Arabian Peninsula, which is characterized by different physiographic conditions, including small regions of deserts (e.g. An Nafud and Ad Dahana), plateaus (Najd), and plains (e.g. Al Hussa and Tabuk-Sirhan-Turayf). Black coat color in the Magaheem dromedaries could be particularly adaptive under the hypothesis that radiation heat load at skin level and the risk of heat stress may be reduced in dark-colored species (Singh et al. 1997; Ward et al. 2002). It was also reported that the Magaheem dromedaries have the highest growth rates and carcass weights among the Arabian Peninsula dromedaries (El-Waziry et al. 2012). It is possible that the *ASIP* gene may have epistatic interaction effects with other genes influencing body weight gains. The *ASIP* could act as an antagonist with other members of the melanocortin receptor family, with important physiological functions (Wolff et al. 1999). For example, the melanocortin 4 receptor (*MC4R*) that plays a role in regulating feeding and metabolism (Cone et al. 1996). The preference of the Bedouins (a desert dwelling community) for large sized animals and unique coat colors might have favored the fixation of the black coat color allele. Also, the diversity of coat colors observed in some dromedary desert populations may be the outcome of Bedouin tribes selecting for hair of different color patterns for making tents and winter clothes whereas the beach and hill dwelling communities typically use stones or wood as the main housing materials.

In conclusion, by comparing the sequence variants of the *MC1R* and *ASIP* genes with the 4 coat color phenotypes found in 10 Arabian Peninsula dromedary populations, we identified candidate polymorphisms that are likely to contribute to coat color variation in the species. First, the light-brown coat color appears to be determined by the proposed wild-type alleles at both genes. Second, the 23^del^T-25A haplotype in the *ASIP* gene is a candidate for the dark pigmentation characteristic of the Magaheem and Sofor dromedary populations. Finally, the 901T allele in *MC1R* is a potential candidate for the white coat color of the Wodh dromedary population. Analysis of more animals with different coat color phenotypes may further strengthen the findings. Moreover, functional and pedigree analyses will be required to further confirm the effect of the mutations identified here on the dromedary coat color variations.

## Supplementary Material

Supplementary material can be found at https://academic.oup.com/jhered/

Supplement Table and Figure

## Funding

This work was supported by King Faisal University, Kingdom of Saudi Arabia.
