# Supplementary material for: Polymorphisms in MC1R and ASIP Genes are Associated with Coat Color Variation in the Arabian Camel
Source: J Hered. 2018 Jun 9;109(6):700–6. doi: 10.1093/jhered/esy024 (PMC6108395; doi:10.1093/jhered/esy024)
Supplement: Supplement Table and Figure [file esy024_suppl_supplement_table_and_figure.docx]

**Supplementary Table S1** Alpaca primer pairs, sequences and the annealing temperatures (Tm) used to amplify the coding regions of the dromedary *MC1R* and *ASIP* genes.

| Gene | Primer name | Sequences | Tm (**°**C) | Source |
| --- | --- | --- | --- | --- |
| *MC1R* | MC1R-Forward | GGGAGAAGGTGAGTGTGAGG | 59 | Feeley & Munyard (2009) |
|  | MC1R-Reverse | GCTCTTCCTGGAGATTCGTG |  |  |
| *ASIP* | Exon2- Forward | CTCAACTGGGACACTTGTGG | 60 | Feeley *et al.* (2011) |
|  | Exon2- Reverse | AGCACAAAGGAGCTGTGACC |  |  |
|  | Exon3- Forward | TCTATTCAGCCAACCCTTCG | 60 |  |
|  | Exon3- Reverse | GGTCTGGTCAGAGCTCAAGG |  |  |
|  | Exon4- Forward | AGAGTCTGAGCAGGTAGTGG* | 65 |  |
|  | Exon4- Reverse | AGGGAGCATGTGCGTAGC |  |  |

* exon 4 the forward primer sequence was modified according to Arabian *Camelus dromedarius* reference genome assembly (GenBank accession number JDVD00000000)

**
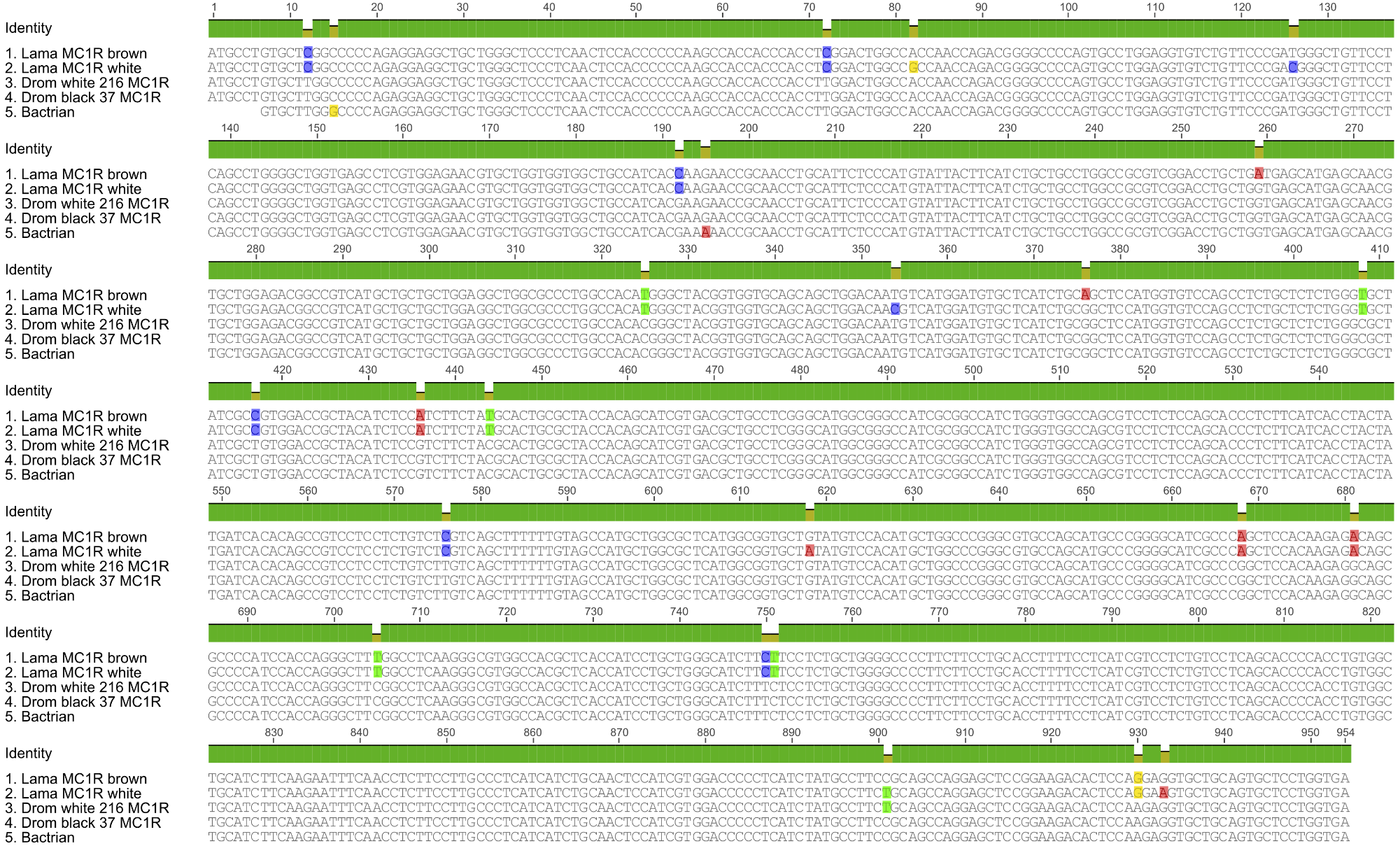
**

**Supplementary Figure S1** Nucleotide sequence comparisons for the *MC1R* exon region for the dromedaries and other camelids reference sequences.


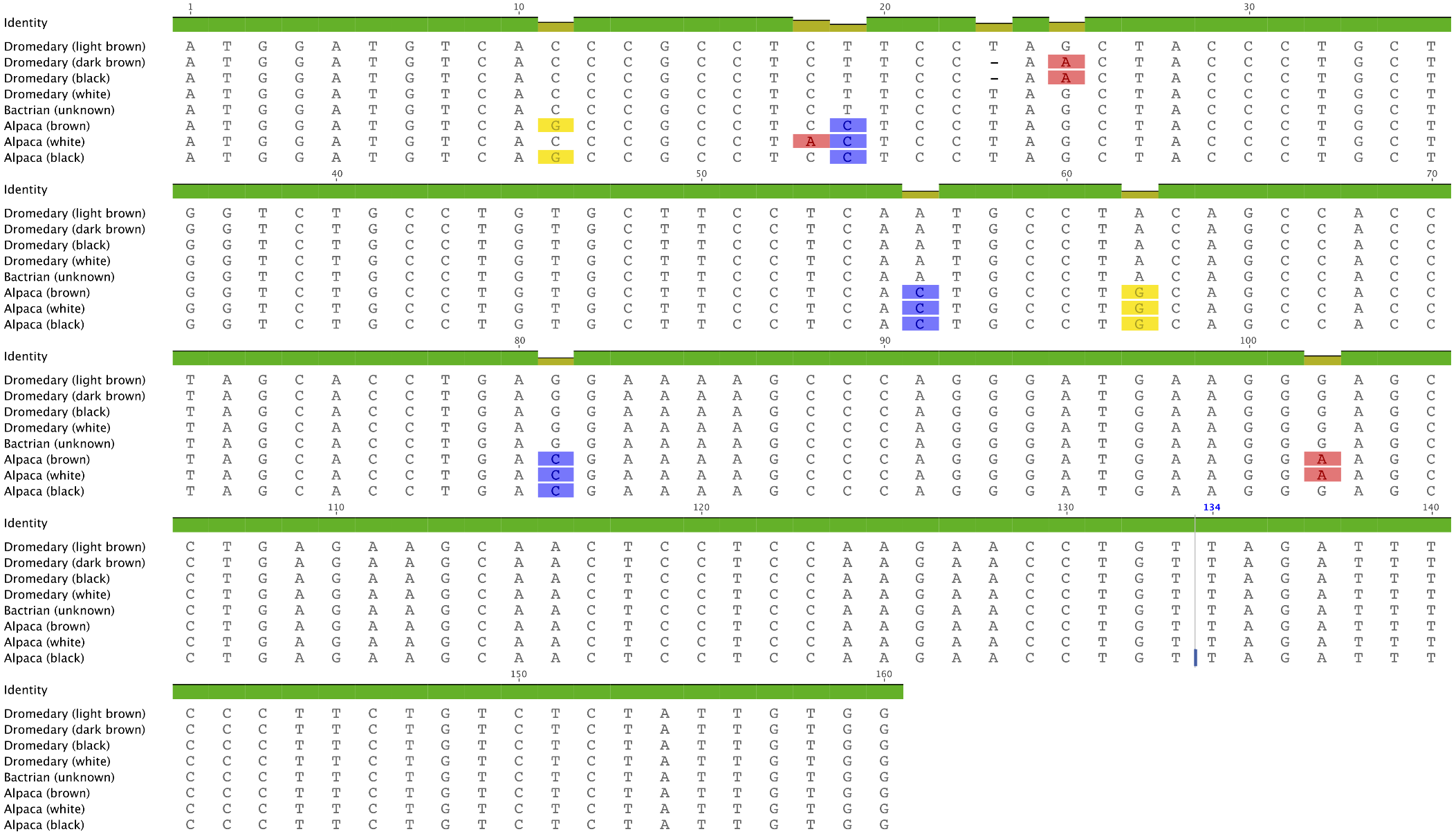


**Supplementary Figure S2** Nucleotide sequence comparisons for the exon 2 of *ASIP* for the dromedary and other camelids reference sequences.

**References**

Feeley NL and Munyard KA 2009. Characterisation of the melanocortin-1 receptor gene in alpaca and identification of possible markers associated with phenotypic variations in colour. Animal Production Science 49, 675-681.

Feeley NL, Bottomley S and Munyard KA 2011. Three novel mutations in ASIP associated with black fibre in alpacas (Vicugna pacos). The Journal of Agricultural Science 149, 529-538.
